# The oncologic safety and accuracy of indocyanine green fluorescent dye marking in securing the proximal resection margin during totally laparoscopic distal gastrectomy for gastric cancer: a retrospective comparative study

**DOI:** 10.1186/s12957-022-02494-5

**Published:** 2022-01-28

**Authors:** Byung Woo Yoon, Woo Yong Lee

**Affiliations:** 1 Department of Internal Medicine, Inje University Seoul Paik Hospital, Jung-gu, Seoul, 04551 Republic of Korea; 2 Inje University College of Medicine, Busan, Republic of Korea; 3 Department of Surgery, Inje University Haeundae Paik Hospital, 875 Haeunda-ro, Haeundae-gu, Busan, 48108 Republic of Korea

**Keywords:** Indocyanine green fluorescence, Operation time, Propensity score matching, Proximal resection margin, Totally laparoscopic distal gastrectomy

## Abstract

**Background:**

Securing the proximal resection margin in totally laparoscopic distal gastrectomy for gastric cancer is related to curability and recurrence, while reducing the operation time is related to patient safety. This study aimed to investigate the role of indocyanine green (ICG) fluorescent dye marking in totally laparoscopic distal gastrectomy, whether it is an oncologically safe and accurate procedure that can be conducted in a single centre.

**Methods:**

The data of 93 patients who underwent laparoscopic-assisted distal gastrectomy (non-ICG group) or totally laparoscopic distal gastrectomy using ICG (ICG group) between 2010 and 2020 were retrospectively reviewed. To correct for confounding factors, a propensity score matching was performed.

**Results:**

Proximal resection margin did not vary with the ICG injection site after the propensity score matching (lower ICG, 3.84 cm vs. lower non-ICG, 4.42 cm, *p* = 0.581; middle ICG, 3.34 cm vs. middle non-ICG, 3.20 cm; *p* = 0.917), while the operation time was reduced by a mean of 34 min in the ICG group (ICG, 239.3 [95% confidence interval, 220.1–258.5 min]; non-ICG, 273.0 [95% confidence interval, 261.6–284.4] min; *p* = 0.006).

**Conclusions:**

ICG injection for securing the proximal resection margin in totally laparoscopic distal gastrectomy is an oncologically safe and accurate procedure, with the advantage of reducing the operation time of gastric cancer surgery while it has the benefit of locating the tumour or clips when it is impossible to locate the tumour during surgery due to the inability to perform an endoscopic examination or when it is hard to directly palpate the tumour or clips in the operating theatre; this can be performed at a single centre.

**Supplementary Information:**

The online version contains supplementary material available at 10.1186/s12957-022-02494-5.

## Background

The standard surgery for gastric cancer requires a D2 lymph node dissection and an appropriate proximal resection margin (PRM) [1]. While most gastric cancer operations are being converted to total laparoscopic gastrectomy, the main important thing is to secure PRM and perform D2 lymph node dissection.

In the past, laparoscopic gastrectomy was an assisted procedure. The stomach was mobilised laparoscopically, and before the main gastrectomy procedure, the PRM distance was determined by palpation of a clip that was attached on the stomach via gastric endoscopy [2, 3]. After the gastrectomy, if a frozen biopsy of the PRM revealed no tumour remnants, an anastomosis was performed; however, if there was a tumour on the PRM, additional cuts were made to ensure negative resection margins.

However, laparoscopic gastrectomy is now a total laparoscopic procedure and the main challenge with this procedure is identifying the tumour location, especially for gastric cancer without serosal invasion or tumours located in the middle-third portion of the stomach, given that the surgeon has secondary contact with the tumour via the laparoscopic equipment and not direct contact.

A secured PRM in gastric cancer is related to curability and recurrence: this is the most important aspect of oncological safety. Currently, in laparoscopic gastric cancer surgeries, indocyanine green (ICG) fluorescence imaging is used to detect and dissect sentinel lymph nodes and confirm blood flow [4–7]. Furthermore, ICG injection is used for tumour localisation during laparoscopic colorectal surgery and real-time surveillance of surgical margins with oral squamous cell carcinoma [8, 9].

In this era of totally laparoscopic distal gastrectomy (TLDG) for gastric cancer, the resection location, which determines the PRM, is identified using intraoperative radiographs. A landmark is created by preoperative endoscopic clipping or tattooing, or intraoperative endoscopy [2, 10, 11]. This increases the operation time (OPT), leading to a longer hospital stay and poor quality of life for the patient [12, 13].

Hence, methods to reduce time and effort during surgery while maintaining a sufficient PRM is required for TLDG. It has been shown that ICG fluorescent dye marking (ICG injection using near-infrared fluorescence) helps surgeons in determining the resection location when palpitation is not possible in the operation room, providing a sufficient PRM, and shortening the operative time [14]. This study aimed to investigate the role of ICG fluorescent dye marking in TLDG, whether it is an oncologically safe and accurate procedure that can be conducted in a single centre.

## Methods

### Study population

**Fig. 1:**
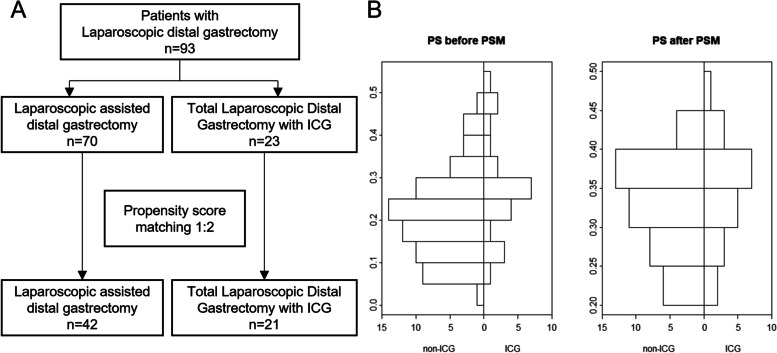
Patient enrolment process. Ninety-three patients with gastric cancer who underwent laparoscopic gastrectomy were identified: 23 underwent laparoscopic distal gastrectomy with indocyanine green (ICG group) and 70 underwent laparoscopic-assisted distal gastrectomy (non-ICG group) (**A**). After the propensity score matching (PSM), 63 patients were enrolled. The patient characters have and uneven distribution between both groups; however, after PSM, there were no significant differences in the ICG and non-ICG groups (**B**)

This retrospective study was approved by the Institutional Review Board of Inje University, Seoul Paik Hospital (2012-05-002-001). Patients with early-stage or locally advanced gastric cancer who underwent a laparoscopic-assisted distal gastrectomy (LADG) or TLDG between January 2010 and November 2020 were enrolled. The patient selection flow chart is shown in Fig. 1A. ICG was used during TLDG to assess and resect the proximal margin; hence, the TLDG group was considered as the ICG group and the LADG group as the non-ICG group. A single operator performed LADG from 2010 to 2017 and TLDG from 2018 to 2020. Although there were no surgical decision criteria on choosing TLDG or LADG, the surgeon decided to switch to TLDG after performing various surgeries difficult for LADG: those with a high body mass index (over 30) or those with a high tumour location. These cases were difficult to perform extra-corporal gastrectomy and gastrojejunostomy with a mini-laparotomy. Hence, after TLDG was endorsed and reimbursed by the Ministry of Health and Welfare of Korea, the surgeon decided to perform TLDG.

The patients’ clinical information (age, sex, gastric cancer staging according to the American Joint Committee on Cancer [AJCC] 8th edition [15], tumour size, surgical method, histologic type, vascular invasion, lymphatic invasion, neural invasion, Lauren’s classification [16], tumour location, PRM, number of dissected lymph nodes, number of positive lymph nodes, and skin-to-skin OPT) was retrospectively reviewed using electronic medical records. Patients who received chemotherapy or radiotherapy before surgery were excluded.

### Surgical protocol

**Fig. 2:**
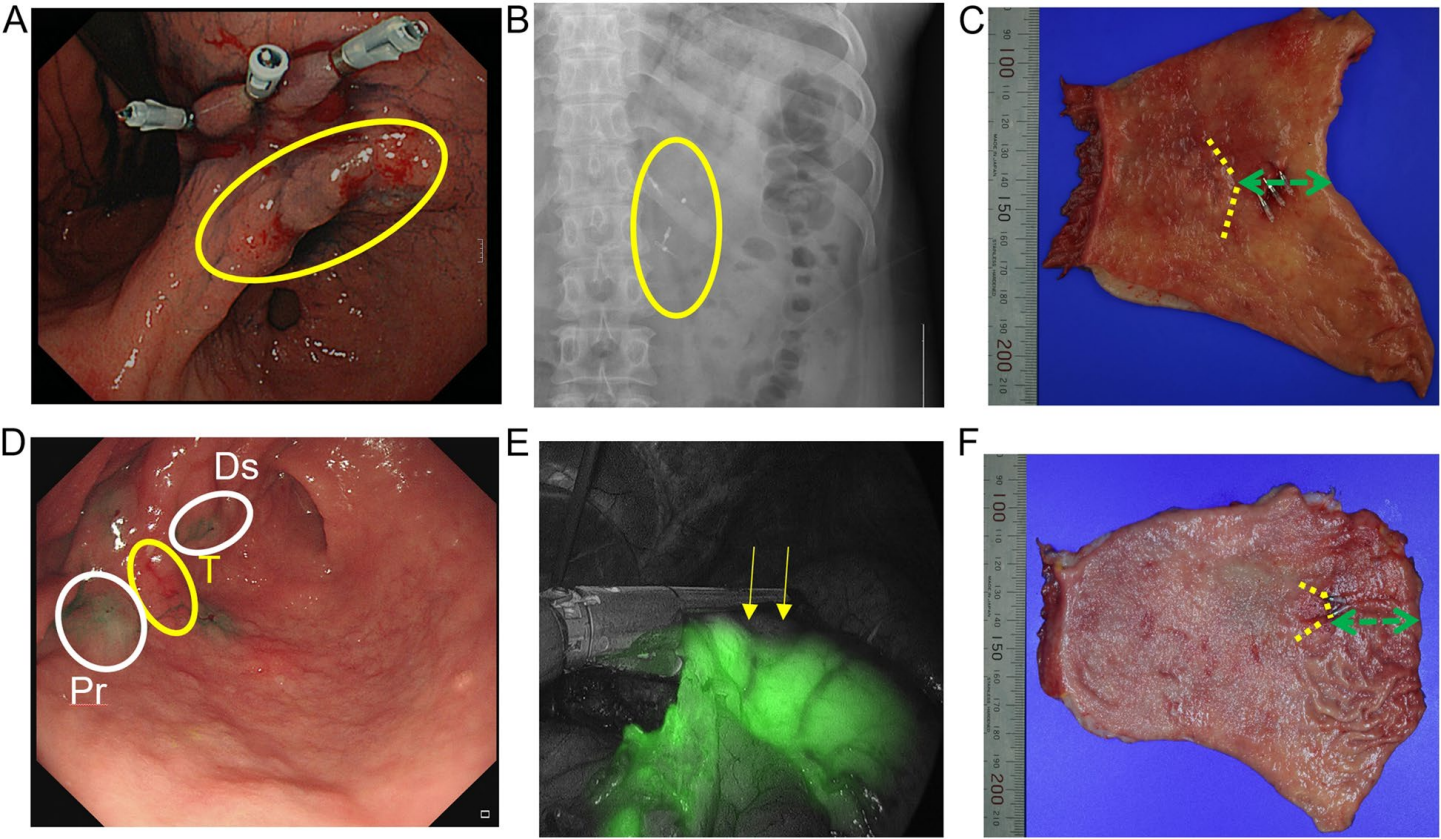
Surgical procedure for proximal margin resection. During laparoscopically assisted gastrectomy (non-ICG group), a clip was inserted 2 cm proximally from the tumour in the yellow circle (**A**), which was verified using intraoperative abdominal radiography, shown in white markers in the yellow circle (**B**). The proximal resection margin (PRM) is indicated by the green arrow, for the non-ICG group, and the tumour border by the dashed yellow line (**C**). For totally laparoscopic-assisted gastrectomy (ICG group), on the day before surgery, 0.1 mL of ICG was injected submucosally in 4 sites (proximal part (Pr in white), distal part (Ds in white), and both lateral parts) around the tumour (T in yellow) (**D**). The camera light was changed to ICG mode, to reveal the proximal margin shown in green with yellow arrows representing the border. An EndoGIA^TM^ stapler was used to cut the PRM directly (**E**). The PRM is indicated by the green arrow, for the ICG group, and the tumour border by the dashed yellow line (**F**)

Regarding the localisation method in the non-ICG group, gastroduodenoscopy was performed the day before surgery. During this procedure, a clip was inserted 2 cm proximally to the tumour (Fig. 2A, tumour marked in yellow circle): this location was identified 1 day before the surgery using abdominal radiography (Fig. 2B, radiopaque clips in the yellow circle). A small incision (mini laparotomy) was made in the epigastric area, and the location of the clips was checked via palpation by the surgeon. Then, a distal gastrectomy was performed above the clips, and the stomach was removed. After confirming the absence of a tumour remnant on the frozen biopsy for the PRM, an extra-corporeal gastrojejunostomy was performed.

In the ICG group, 1 day before surgery, 0.1 mL of ICG (indocyanine green, Donggindang Pharmaceutical Co, Siheung, Korea; 2.5 mg of ICG was dissolved in 5 mL of distilled water) was injected submucosally in 4 locations (proximal part, distal part, and both lateral parts) approximately within 1 cm from the borders of the tumour (Fig. 2D), resulting in a total of 0.4 mL of ICG [17, 18]. Clipping was performed on the proximal part of the tumour (Fig. 2D, clips in the circle). Before gastrectomy, the camera view was changed to ICG mode during the laparoscopic procedure to reveal the proximal margin. Gastrectomy was performed using a Medtronic EndoGIA^TM\^ stapler (Fig. 2E, proximal margin seen green, and borders marked by yellow arrows). Once the specimen was removed, intra-corporeal gastrojejunostomy was performed after the frozen biopsy of the PRM was confirmed to be negative for tumour remnants. If there was tumour on the resection margins, further operation was performed until negative tumour margin. The gross anatomy of the stomach is shown in Fig. 2C and F, respectively, where the PRM length is marked by green arrows, which was measured after surgery by the pathologist defined by the shortest distance from the tumour to the PRM.

Subtotal gastrectomy was performed for all gastric resections, with Billroth II gastrojejunostomy (antecolic and isoperistalic gastrectomy) as the anastomosis method. Standard gastrectomy with a D2 resection for lymph nodes was performed for all patients [1]. The OPT was defined as the surgery duration, from the start to the end of the surgery (skin opening to skin closure).

### Statistical analysis

All analyses were performed using R 4.1.0. The *t*-test was used to compare continuous variables between the two groups. For comparisons of categorical variables, Pearson’s chi-square test was used. The *p*-value was calculated using Pearson’s chi-square test from the continuous data comparison ratio. Meanwhile, the *p*-value was calculated using the Kolmogorov–Smirnov (KV) test to compare the continuous distribution of the time density plots. To minimise the differences in baseline characteristics, propensity score matching (PSM) with optimal matching was performed using the *MatchIt* [19] and *optmatch* [20] packages in R. PSM was based on age, sex, AJCC stage for gastric cancer, tumour location, and tumour size; a ratio of 1:2 for the ICG and non-ICG groups was used. Effective size was calculated by Cohen’s *d* for PRM and OPT after PSM [21, 22].

## Results

### Propensity score matching

Of the 93 enrolled patients, 23 were in the ICG group and 70 in the non-ICG group (Fig. 1A). There were differences in the T stage, N stage, and tumour size between the groups (Table 1); therefore, PSM was used to match similar baseline characteristics between the two groups. After the PSM, there were 42 patients in the non-ICG group and 21 patients in the ICG group. There was no significant difference in age, sex, stage, tumour location, and tumour size. The patients’ characteristics before and after PSM are shown in Fig. 1B. The density distribution histograms before PSM for the two groups were different, while those after PSM were similar.

**Table 1:** Clinical characteristics of 93 patients with gastric cancer and its propensity score-matched results

	Before propensity score matching (*n* = 93)			After propensity score matching (*n* = 63)		
Characteristics	Non-ICG (*n* = 70)	ICG (*n* = 23)	*p*-value	Non-ICG (*n* = 42)	ICG (*n* = 21)	*p*-value
Age over 65 (%)	38 (54.3)	10 (43.5)	0.51	21 (50.0)	9 (42.9)	0.79
Gender (%)			0.55			1.000
Male	45 (64.3)	17 (73.9)		29 (69.0)	15 (71.4)	
Female	25 (35.7)	6 (26.1)		13 (31.0)	6 (28.6)	
EGD Result (%)			0.61			1.00
AGC	10 (14.3)	5 ( 21.7)	0.61	8 (19.0)	4 (19.0)	
EGC	60 (85.7)	18 ( 78.3)		34 (81.0)	17 (81.0)	
T (%)			0.34			1.00
T1	60 (85.7)	19 (82.6)		38 (90.5)	19 (90.5)	
T2	9 (12.9)	3 (13.0)		4 ( 9.5)	2 ( 9.5)	
T3	1 ( 1.4)	0 ( 0.0)		–	–	
T4a	0 ( 0.0)	1 ( 4.3)		–	–	
N (%)			0.09			1.00
N0	63 (90.0)	18 (78.3)		37 (88.1)	18 (85.7)	
N1	7 (10.0)	3 (13.0)		6 (11.9)	3 (14.3)	
N3a	0 ( 0.0)	1 ( 4.3)		–	–	
N3b	0 ( 0.0)	1 ( 4.3)		–	–	
Stage (%)			0.03			1.00
I	68 (97.1)	21 (91.3)		41 (97.6)	21 (100.0)	
II	2 ( 2.9)	0 ( 0.0)		1 ( 2.4)	0 ( 0.0)	
III	0 ( 0.0)	2 ( 8.7)				
Location (%)			0.44			0.93
Lower	42 (60.0)	11 (47.8)		22 (52.4)	10 (47.6)	
Middle	28 (40.0)	12 (52.2)		20 (47.6)	11 (52.4)	
LI (%)			1.00			0.53
N	44 (62.9)	14 (60.9)		23 (54.8)	14 (66.7)	
Y	26 (37.1)	9 (39.1)		19 (45.2)	7 (33.3)	
VI (%)			1.00			1.00
N	68 (97.1)	23 (100.0)		42 (100.0)	21 (100.0)	
Y	2 (2.9)	0 ( 0.0)		0 ( 0.0)	0 ( 0.0)	
NI (%)			0.73			1.00
N	59 (84.3)	18 (78.3)		37 (88.1)	18 (85.7)	
Y	11 (15.7)	5 (21.7)		5 (11.9)	3 (14.3)	
Tumor size (cm), mean (SD)	2.76 (1.70)	3.34 (2.95)	0.24	2.56 (1.56)	2.58 (1.58)	0.97
Dissected lymph nodes, mean (SD)	30.54 (10.63)	31.17 (8.21)	0.80	31.33 (11.95)	31.14 (8.43)	0.95
Positive lymph nodes, mean (SD)	0.16 (0.50)	1.43 (4.34)	0.02	0.17 (0.49)	0.14 (0.36)	0.84
Positive node ratio (%), mean (SD)	0.54 (1.75)	4.61 (13.75)	0.02	3.74 (1.56)	3.58 (1.52)	0.81
PRM (cm), mean (SD)	4.11 (1.79)	3.42 (1.60)	0.10	3.74 (1.56)	3.58 (1.52)	0.70
Operation time (min), mean (SD)	270.1 (37.3)	246.1 (46.7)	0.01	273.0 (36.6)	239.3 (42.3)	0.002

*ICG* Indocyanine green, *SD* Standard deviation, *EGD* Esophagogastroduodenoscopy, *LI* Lymphatic invasion, *VI* Vascular invasion, *NI* Neural invasion, *PRM* Proximal resection margin

### Baseline characteristics of patients before and after PSM

The baseline characteristics of the pre- and post-matched cohorts are presented in Table 1. Before PSM, there were differences in age, female/male ratio, T stage, N stage, tumour location, and tumour size between the non-ICG and ICG groups. After PSM, *p*-values increased, indicating no differences of groups, for age above 65 from 0.51 to 0.79, T stages p- from 0.34 to 1.00, N stages from 0.09 to 1.00, location of the tumour from 0.44 to 0.93, and tumour size from 0.24 to 0.97.

### The comparison of PRM between the ICG and non-ICG groups

**Fig. 3:**
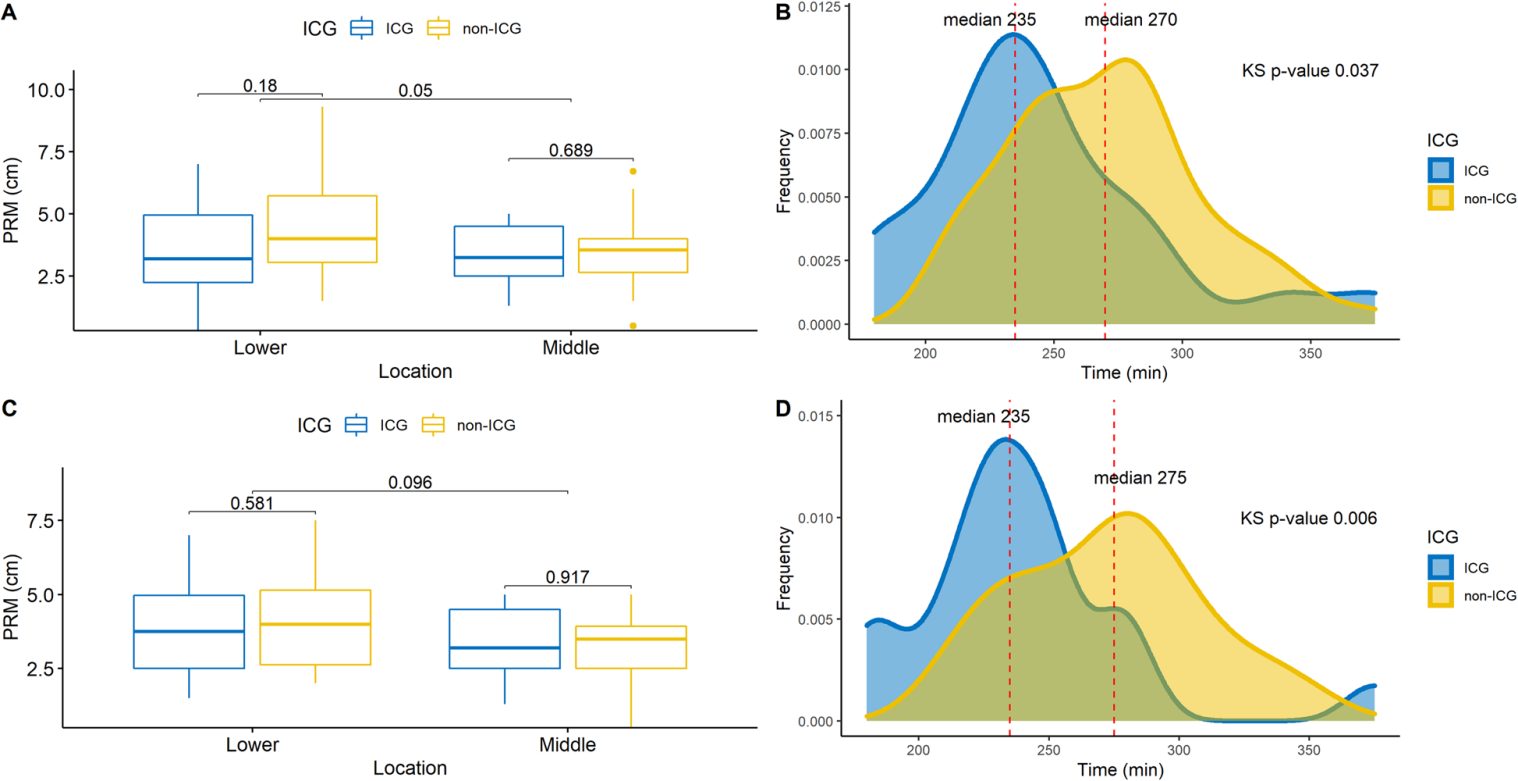
The proximal resection margin (PRM) and operation time before and after the propensity score matching (PSM). There was a slight difference in the PRM between the lower part and the middle part (*p* = 0.05), while there was no difference between ICG or non-ICG groups (**a**). The median operation time for the ICG and non-ICG groups was 235 min and 270 min, respectively (**b**). After PSM, there was no difference in the PRM between the lower part and the middle part. There was also no difference according to ICG injection site (**c**). The median operation time of the ICG and non-ICG groups was 235 min and 275, respectively (*p* = 0.006) (**d**)

Before the PSM, there was a significant difference in PRM according to location (lower PRM mean = 4.32 vs. middle PRM mean = 3.44 cm; *p* = 0.06) (Fig. 3A). However, when comparing PRM in the same location, there was no significant difference in PRM between the ICG and non-ICG groups in the lower or middle section. Although the PRM was smaller in the ICG group than in the non-ICG group, this was not the case after the PSM. There was no significant difference in PRM in the ICG and non-ICG groups in the lower section (ICG PRM mean = 3.84 cm vs. non-ICG PRM mean = 4.22 cm; *p* = 0.581) or in the middle section (ICG PRM mean = 3.34 vs. non-ICG PRM mean = 3.20 cm; *p* = 0.917 (Fig. 3C and Supplementary Table 1).

The effective size was small with a Cohen’s *d* of 0.10 for PRM after PSM, signifying that there is no difference of PSM between the two groups.

### The comparison of surgical time between the ICG and non-ICG groups

There was some difference in the OPT between the ICG and non-ICG groups before PSM. The median OPT for the ICG group was 235 min, while the median OPT for the non-ICG group was 270 min with a KS *p*-value of 0.037 (Fig. 3B).

After PSM, the difference in the OPT between both groups increased. The median OPT for the ICG group was 235 min, while the median OPT for the non-ICG group was 275 min, with a KS *p*-value of 0.006 (Fig. 3D).

In addition, the effective size was large with a Cohen’s *d* of 0.87, signifying that OPT time is different between the two groups.

## Discussion

Securing the PRM is necessary for curative gastric cancer surgery. According to the Japanese gastric cancer surgery guidelines, at least 2 cm and 3–5 cm should be secured during early gastric cancer and advanced gastric cancer surgeries, respectively [1]. Squires et al. [23] argued that there was no difference in survival outcomes of early gastric cancer with a PRM of 3 cm or 5 cm and that other factors influence survival outcomes of advanced gastric cancer. However, Ha et al. [24] and Ohe et al. [25] suggested that PRM may be related to prognosis in advanced gastric cancer and recommended a PRM of at least 3 cm in controversial situations [5, 8, 10, 11].

With the increasing use of TLDG, the main challenge is identifying the exact tumour location in the laparoscopic field of view, especially for tumours without serosal invasion. This is difficult because the tactile force of the laparoscopic arm cannot provide tactile stimulation, given its direct contact with the tumour. The PRM can be confirmed using various methods. Two methods were used in this study: preoperative endoscopic clipping and ICG injection. Specifically, we investigated whether the use of ICG injection is a good method to secure PRM in TLDG, although currently, the use of ICG to detect and dissect sentinel lymph nodes in gastric cancer is recommended [26, 27].

Upper endoscopy tattooing procedures is similar to this method; however, ICG dye injection is a more reliable method of operation as this provides a clear view of the PRM during laparoscopy, whereas tattooing only specifies the tumour border by the dye, which may be at the risk of not being shown, especially during laparoscopy [28, 29].

In this study, the mean PRM was 3.6 cm and 3.7 cm (*p* = 0.70) in the ICG and non-ICG groups, respectively (Table 1). This is similar to the results of Ushimaru et al. [14], where there was no significant difference in the PRM: 5.7 cm and 5.4 cm (*p* = 0.433) for the ICG and non-ICG group, respectively. Although the PRM was smaller in the ICG group in our study after PSM (Supplementary Table 1), it was not significant (Fig. 3A, Fig. 3C), and a small effect size of using Cohen’s *d* shows that a value of 0.10 shows that there is no effect of ICG or non-ICG group in determining PRM. As indicated in the Japanese guidelines [1] and studies by Ha et al. [24] and Ohe et al. [25], a PRM above 3 cm is sufficient.

While some may argue that there may be no utility regarding lower-section PRM, we have conducted this as well as middle-section PRM as minimising the PRM will eventually increase the functional residual volume, which leads to better functional outcomes [30]. However, there were no statistically significant reductions of the PRM for the lower section as well as the middle section.

Regarding the ICG injection safety, there are no consensus on the amount of ICG dye required. In our case, we used 0.1 mL of 0.5 mg/mL concentrated ICG dye before the day of operation, which is concordant to the protocol suggested by Yoshida et al. [17]. This amounts to a total of 0.2 mg of ICG dye for 4 sites. In this concentration, no patient reported any clinical side effect, and none was observed on the injection site. Furthermore, during the operation, there was no failure of fluorescence from the ICG when the camera view was changed to ICG mode. Interestingly, no tumour was found in the frozen biopsy from the PRM in the ICG group. This is the main benefit of ICG injection, compared to LADG in PRM determination. In TLDG, the operator can save time in determining the PRM using intraoperative radiography, laparotomy, and palpation of the clips. To secure the PRM, a tattoo of 3–5 cm is made from the preoperative endoscopic clippings; this procedure is laborious and makes the operator less confident, given that if tumour remnants are identified on the frozen biopsy of the PRM, addition resection is required. This can be done without additional equipment or additional personnel, enabling its use in a single centre.

The main limitation of this study was its uneven distribution of ICG and non-ICG sample size and retrospective nature. However, this was overcome using the PSM to reduce covariance and bias. After the PSM, the patient characteristics were matched, allowing for a fair comparison, where there was no difference in PRM between the ICG and non-ICG groups, while the difference in OPT reduced significantly in the ICG group. This suggests the usefulness of ICG injection in labour and time reduction for the surgeon, increase in patient safety, and reduction of the total OPT, leading to a rapid recovery for discharge. Although Fogarty et al. [31] argues that prolonged operation time of over 6 hours is not the main contributor of postoperative complications in most surgeries, Jackson et al. [12] suggested that increased operational time is associated with increased odds of complications in laparoscopic surgery. In addition, in the multivariate analysis of risk factors in postoperative complication after laparoscopic gastrectomy, Hyon et al. [13] showed that the odds ratio was significantly higher if the operation lasted over 240 min. In our study, the distribution of OPT for ICG group was within 235 min, while the non-ICG was near 275 min, where this time difference may be an important factor in postoperative complications.

The differences in OPT may be due to the innovation of devices and techniques of the operator during the long practice period. We also do admit that we should have timed the tumour to resection-anastomosis time only to see the merit of TLDG. However, as LADG requires meticulous palpation to check the clips, or radiography of the clips when unpalpable, LADG has less benefit in terms of total OPT compared to TLDG, where the surgeon only needs confidence that ICG dye will provide sufficient PM. An analysis on yearly OPT is plotted in the supplementary Fig. 1A, which shows that in the non-ICG group, there is no time benefit from the learning curve: OPT diverges due to the difficulty of the operation, which was proved with PSM in the supplementary Fig. 1B. However, in the ICG group, there is a time benefit from the learning curve.

## Conclusions

ICG injection in TLDG improved the OPT and had a similar PRM, when compared with LADG. Furthermore, this has a benefit of locating the tumour or clips when it is impossible to locate the tumour during surgery due to the inability to perform an endoscopic examination or when it is hard to directly palpate the tumour or clips in the operating theatre. Hence, ICG injection in TLDG is an oncologically safe and accurate procedure that can be performed in a single centre.

## Supplementary Information

**Additional file 1: Supplementary Fig. 1.** Operation time of each year. The operation time for ICG and non-ICG group before (A) and after propensity score matching (B). The initial operation time for non-ICG group and ICG group is similar. As can be seen in both graphs, the operation time for non-ICG does not reduce with surgical experience. However, in the ICG group, there is an operation time reduction with surgical experience.

**Additional file 2. Supplementary Table 1. **The proximal resection margins before and after propensity score matching.

## Data Availability

Not applicable

## Abbreviations

PRM: Proximal resection margin
TLDG: Totally laparoscopic distal gastrectomy
OPT: Operation time
ICG: Indocyanine green
PSM: Propensity score matching
LADG: Laparoscopic-assisted distal gastrectomy
