# Supplementary figures and images for: The oncologic safety and accuracy of indocyanine green fluorescent dye marking in securing the proximal resection margin during totally laparoscopic distal gastrectomy for gastric cancer: a retrospective comparative study
Source: World J Surg Oncol. 2022 Jan 28;20:26. doi: 10.1186/s12957-022-02494-5 (PMC8796580; doi:10.1186/s12957-022-02494-5)

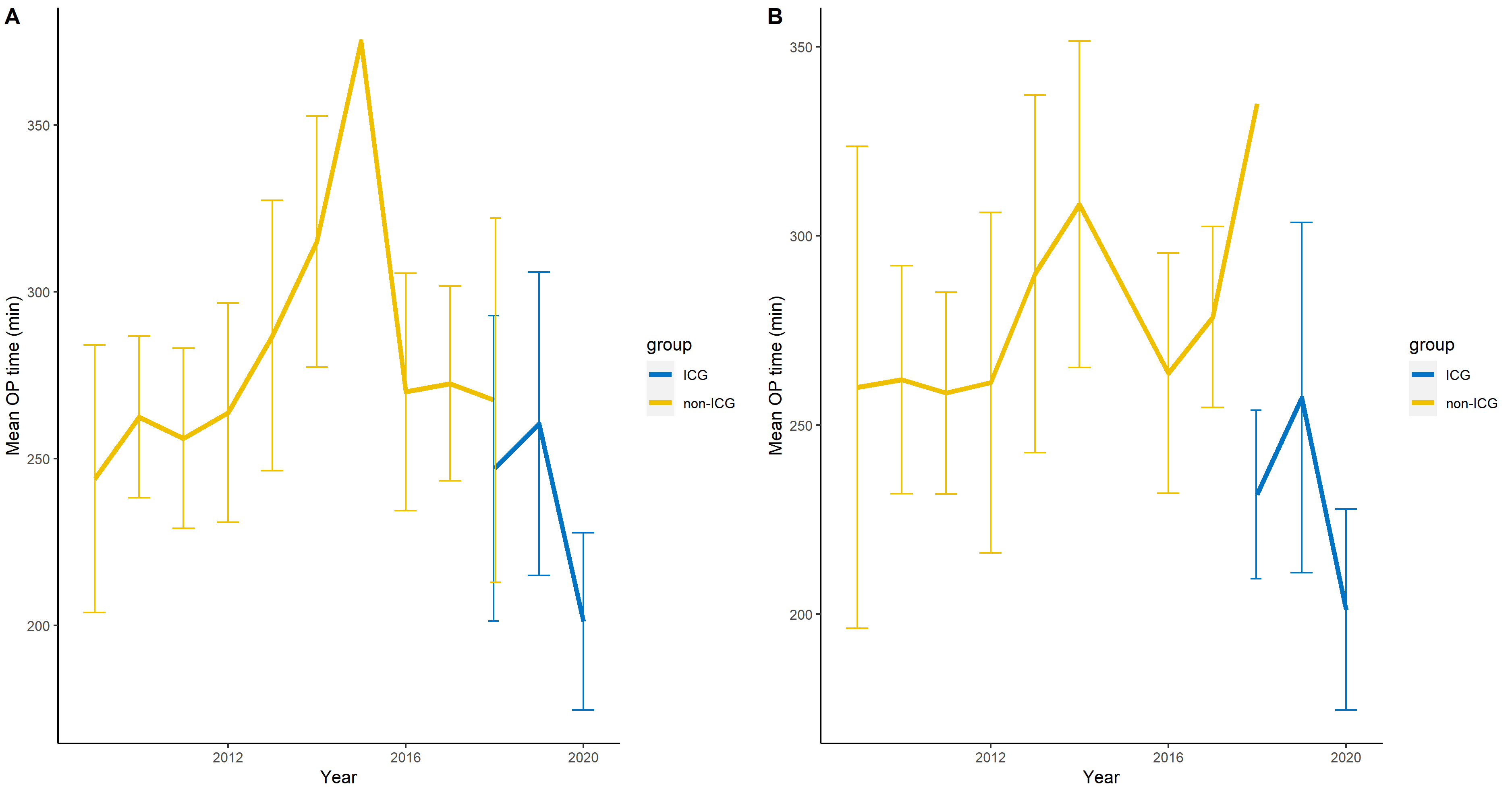

Supplement: Supplementary file 1 — Additional file 1: Supplementary Fig. 1. Operation time of each year. The operation time for ICG and non-ICG group before (A) and after propensity score matching (B). The initial operation time for non-ICG group and ICG group is similar. As can be seen in both graphs, the operation time for non-ICG does not reduce with surgical experience. However, in the ICG group, there is an operation time reduction with surgical experience. [file 12957_2022_2494_MOESM1_ESM.png]
