# Supplementary material for: The oncologic safety and accuracy of indocyanine green fluorescent dye marking in securing the proximal resection margin during totally laparoscopic distal gastrectomy for gastric cancer: a retrospective comparative study
Source: World J Surg Oncol. 2022 Jan 28;20:26. doi: 10.1186/s12957-022-02494-5 (PMC8796580; doi:10.1186/s12957-022-02494-5)
Supplement: Supplementary file 2 — Additional file 2. Supplementary Table 1. The proximal resection margins before and after propensity score matching. [file 12957_2022_2494_MOESM2_ESM.docx]

**Supplementary Table 1. The proximal resection margins before and after propensity score matching**

| Location | ICG | PRM mean (cm) [95% CI] before PS match | PRM mean [95% CI]  after PS match |
| --- | --- | --- | --- |
| Lower | ICG | 3.52 [2.18~4.86] | 3.84 [2.57~5.11] |
|  | Non-ICG | 4.54 [3.92~5.13] | 4.22 [3.44~4.99] |
| Middle | ICG | 3.33 [2.55~4.11] | 3.34 [2.47~4.20] |
|  | Non-ICG | 3.49 [2.99~4.00] | 3.20 [2.67~3.74] |

ICG: Indocyanine green fluorescence with totally laparoscopic gastrectomy, non-ICG: laparoscopic assisted distal gastrectomy with clipping, PS: propensity score
